# Crystal structure of the adduct (4-chloro­phen­yl)(4-hy­droxy­piperidin-1-yl)methanone–(4-chloro­phen­yl)(piperidin-1-yl)methanone (0.75/0.25)

**DOI:** 10.1107/S2056989015020265

**Published:** 2015-10-31

**Authors:** B. K. Revathi, D. Reuben Jonathan, K. Kalai Sevi, K. Dhanalakshmi, G. Usha

**Affiliations:** a PG and Research Department of Physics, Queen Mary’s College, Chennai-4, Tamilnadu, India; b Department of Chemistry, Madras Christian College, Chennai-59, India; c SCRI, Anna Hospital Campus, Chennai-106, Tamilnadu, India; d Anna Siddha Medical College, Chennai-106, Tamilnadu, India

**Keywords:** crystal structure, adduct, piperidine derivative, hydrogen bonding

## Abstract

In the title compound, 0.75C_12_H_14_ClNO_2_·0.25C_12_H_14_ClNO, which is an adduct comprising 0.75 4-hy­droxy­piperidin-1-yl or 0.25 4-piperidin-1-yl substituents on a common (4-chloro­phen­yl)methanone component; the dihedral angles between the benzene ring and the two piperidine rings are 51.6 (3) and 89.5 (7)°, respectively. The hy­droxy­piperidine ring is in a bis­ectional oriention (*bi*) with the phenyl ring. In the crystal, inter­molecular O—H⋯O hydrogen bonds between the hy­droxy­piperidine group and the keto O atom lead to the formation of chains extending along the *c*- axis direction.

## Related literature   

For the synthesis, see: Revathi *et al.* (2015 ▸). For the biological activity of piperidine derivatives, see: Ramalingan *et al.* (2004 ▸); Sargent & May (1970 ▸); Rubiralta *et al.* (1991 ▸). For related structures, see: Revathi *et al.* (2015 ▸); Prathebha *et al.* (2015 ▸).
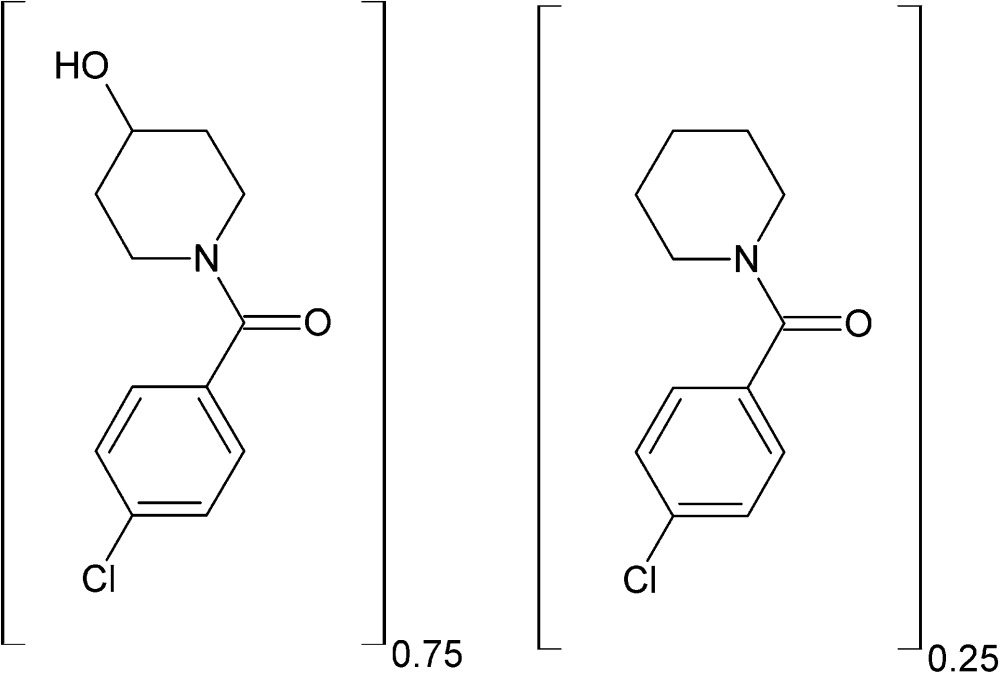


## Experimental   

### Crystal data  

0.75C_12_H_14_ClNO_2_·0.25C_12_H_14_ClNO
*M* *_r_* = 235.69
Orthorhombic, 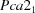

*a* = 24.312 (4) Å
*b* = 6.1628 (10) Å
*c* = 7.9654 (11) Å
*V* = 1193.5 (3) Å^3^
*Z* = 4
Mo *K*α radiation
μ = 0.30 mm^−1^
*T* = 293 K
0.25 × 0.20 × 0.20 mm

### Data collection  

Bruker Kappa APEXII CCD diffractometer
Absorption correction: multi-scan (*SADABS*; Bruker, 2004 ▸) *T*_min_ = 0.930, *T*_max_ = 0.941
17321 measured reflections
2356 independent reflections
1539 reflections with *I* > 2σ(*I*)
*R*_int_ = 0.031

### Refinement  

*R*[*F* ^2^ > 2σ(*F* ^2^)] = 0.048
*wR*(*F* ^2^) = 0.144
*S* = 1.02
2356 reflections
200 parameters
121 restraints
H-atom parameters constrained
Δρ_max_ = 0.31 e Å^−3^
Δρ_min_ = −0.22 e Å^−3^
Absolute structure: Flack *x* determined using 583 quotients [(*I* ^+^)−(*I* ^−^)]/[(*I* ^+^)+(*I* ^−^)] (Parsons *et al.*, 2013 ▸)
Absolute structure parameter: 0.03 (3)

### 

Data collection: *APEX2* (Bruker, 2004 ▸); cell refinement: *APEX2* and *SAINT* (Bruker, 2004 ▸); data reduction: *SAINT* and *XPREP* (Bruker, 2004 ▸); program(s) used to solve structure: *SIR92* (Altomare *et al.*, 1993 ▸); program(s) used to refine structure: *SHELXL2014* (Sheldrick, 2015 ▸); molecular graphics: *ORTEP-3 for Windows* (Farrugia, 2012 ▸) and *Mercury* (Bruno *et al.*, 2002 ▸); software used to prepare material for publication: *SHELXL2014*.

## Supplementary Material

Crystal structure: contains datablock(s) I, New_Global_Publ_Block. DOI: 10.1107/S2056989015020265/zs2348sup1.cif

Structure factors: contains datablock(s) I. DOI: 10.1107/S2056989015020265/zs2348Isup2.hkl

Supporting information file. DOI: 10.1107/S2056989015020265/zs2348Isup3.cml

**Fig. 1.**
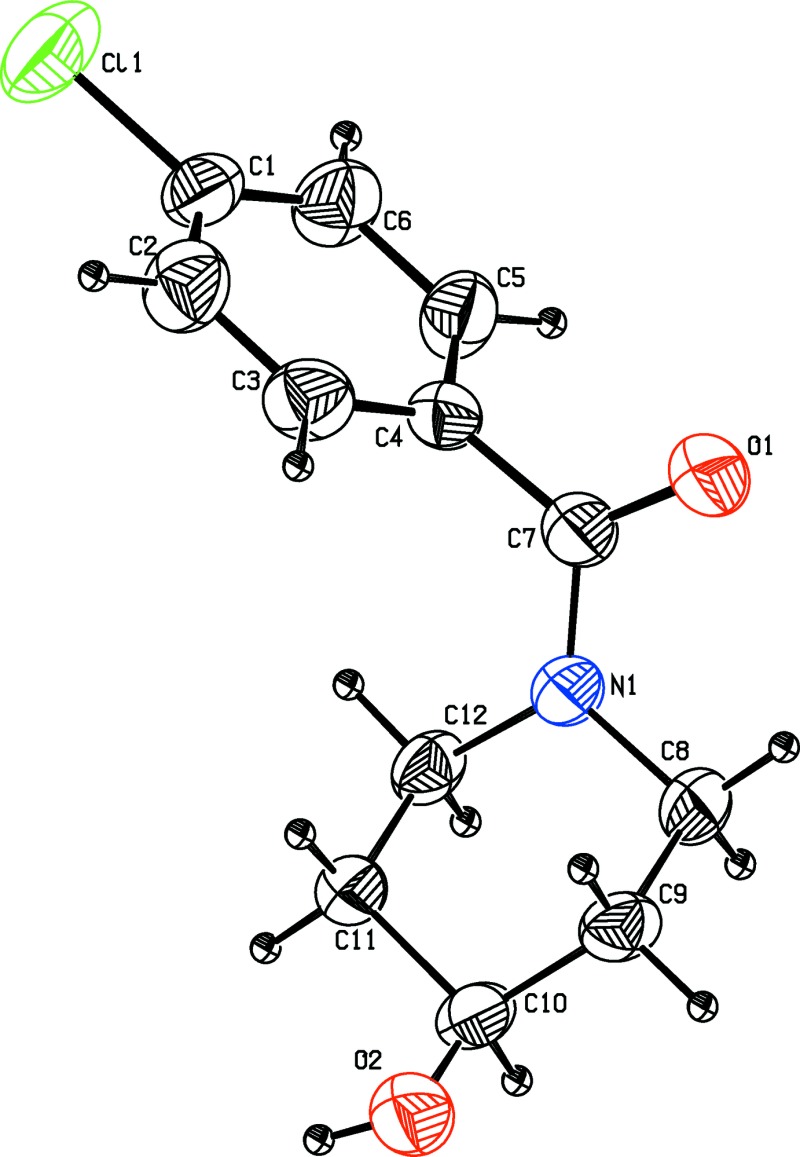
The molecular structure of the major (73%) 4-hydroxypiperidin-1-yl substituted component of the title adduct, showing atom numbering, with displacement ellipsoids drawn at the 30% probability level.

. DOI: 10.1107/S2056989015020265/zs2348fig1.tif
The mol­ecular structure of the major (73%) 4-hy­droxy­piperidin-1-yl substituted component of the title adduct, showing atom numbering, with displacement ellipsoids drawn at the 30% probability level.

**Fig. 2.**
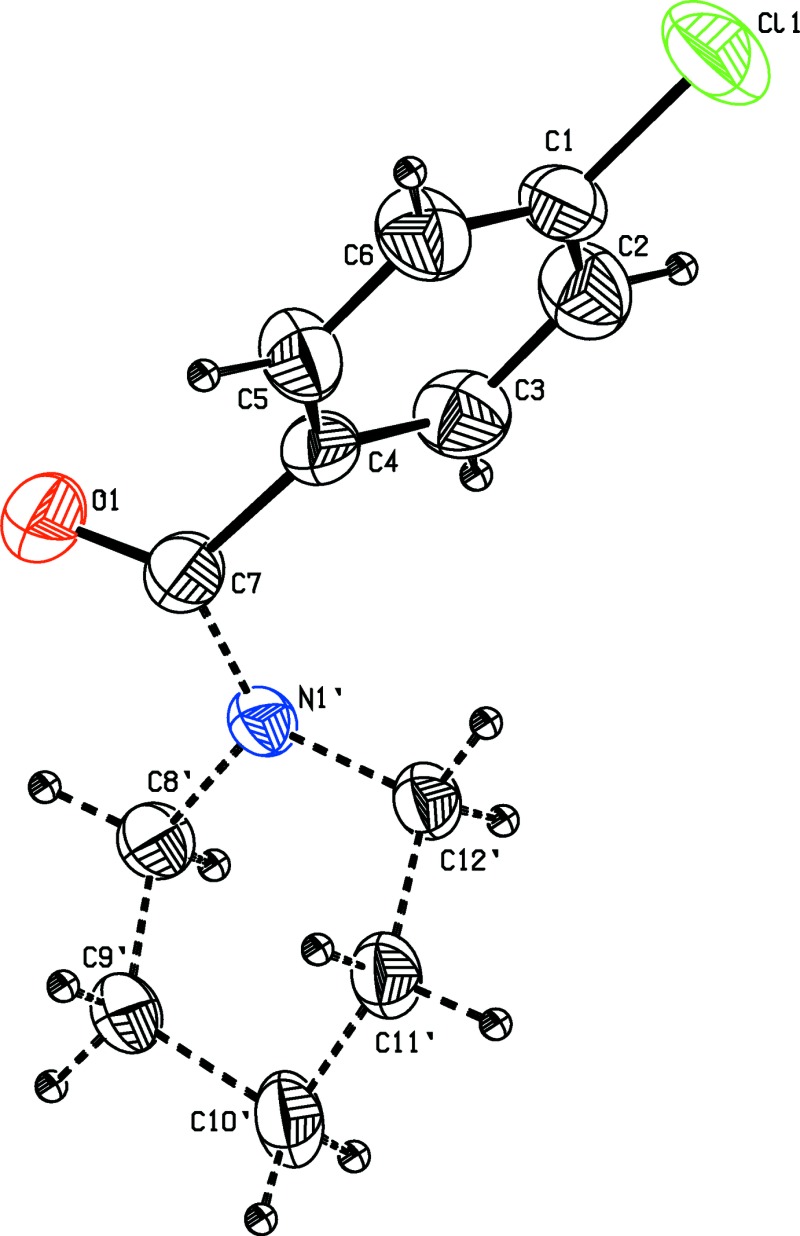
The molecular structure of the minor (25%) piperidin-1-yl substituted component of the title adduct, showing atom numbering, with displacement ellipsoids drawn at the 30% probability level. The bonds for the minor-occupancy piperidinyl group are shown as dashed lines

. DOI: 10.1107/S2056989015020265/zs2348fig2.tif
The mol­ecular structure of the minor (25%) piperidin-1-yl substituted component of the title adduct, showing atom numbering, with displacement ellipsoids drawn at the 30% probability level. The bonds for the minor-occupancy piperidinyl group are shown as dashed lines

**Fig. 3.**
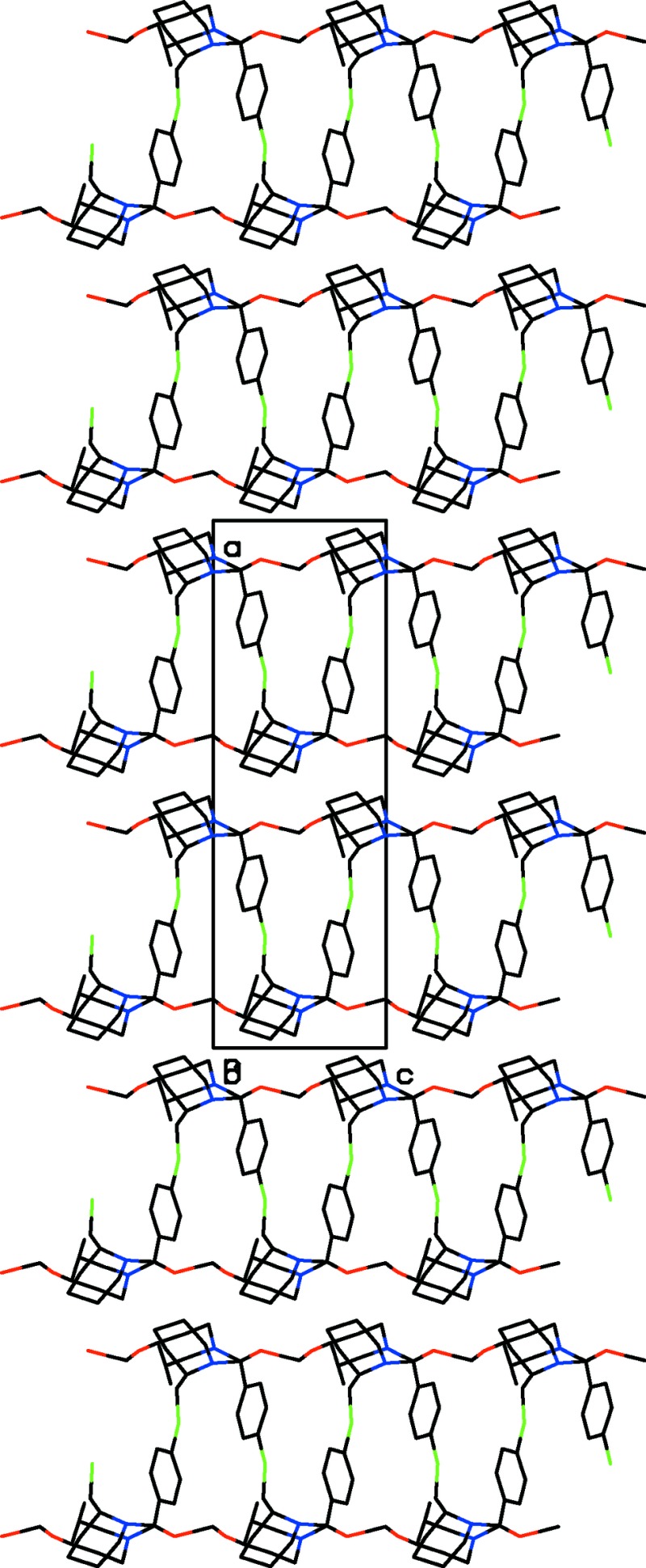
The packing of the molecules in the crystal structure. The dashed lines indicate the O—H···O hydrogen bonds and weak inter-chain C—H···Cl interactions.

. DOI: 10.1107/S2056989015020265/zs2348fig3.tif
The packing of the mol­ecules in the crystal structure. The dashed lines indicate the O—H⋯O hydrogen bonds and weak inter-chain C—H⋯Cl inter­actions.

CCDC reference: 1433393

Additional supporting information:  crystallographic information; 3D view; checkCIF report

## Figures and Tables

**Table 1:** Hydrogen-bond geometry (, )

*D*H*A*	*D*H	H*A*	*D* *A*	*D*H*A*
O2H2*A*O1^i^	0.82	2.05	2.693(7)	135

Symmetry code: (i) 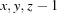.

## supplementary crystallographic information

### S1. Comment

Piperidine is very important pharmacophore because of its presence in numerous alkaloids, pharmaceuticals, agrochemicals and as synthetic intermediates. Biologically active alkaloids with substituted piperidine ring systems have been targeted for their total or partial synthesis (Ramalingan *et al.*, 2004). Piperidines are known to have CNS depressant action at low dosage levels and stimulant activity with increased dosages. In addition, the nucleus also possesses analgesic, ganglionic blocking and anesthetic properties as well (Sargent & May, 1970; Rubiralta *et al.*, 1991).

In the title compound, 0.75(C_12_H_14_NO_2_Cl) . 0.25(C_12_H_14_NOCl), which is an adduct comprising 0.75(4-hydroxypiperidin-1-yl) or 0.25(4-piperidin-1-yl) substituents on a common (4-chlorophenyl)methanone component, the dihedral angles between the benzene ring and the two piperidine rings defined by N1–C12 and N1'–C12' are 51.6 (3) and 89.5 (7)°, respectively (Figs. 1, 2). The C—C distances in the hydroxypiperidine ring and the benzene ring are in the range [1.472 (9)–1.529 (8)Å and 1.346 (7)–1.390 (8)Å], respectively and are in good agreement with literature values (Allen *et al.*, 1987). The C—N distances are in the range [1.346 (7)Å - 1.465 (7)Å] and are in good agreement with values in a similar reported structure (Revathi *et al.*, 2015). The C—O distance [1.223 (6)Å] indicates double bond character and is comparable with the value reported previously (Prathebha *et al.*, 2015). The hydroxypiperdine ring is in a bisectional oriention (*bi*) with the phenyl ring. The sum of the bond angle around the N1 atom is [359.9 (5)°], showing sp^2^ hybridization of the atoms. The torsion angle C8—N1—C7—O1 [12.3 (10)°], indicates that the keto group is in a +*syn-periplanar* (+*sp*) orientation with the hydroxy piperidine ring. The hydroxypiperidine ring adopts a chair conformation with puckering parameters of q2 = 0.029Å, phi2 = -173.57° q3 = -0.555Å, QT = 0.555Å and theta2 = 176.95°.

In the crystal, molecules are linked by O2—H···O1^i^ hydrogen bonds (Table 1), forming one-dimensional chains extending along *c* (Fig. 3). Present also are very weak inter-chain C12'—H···Cl1^ii^ interactions [3.63 (2) Å]. For symmetry code (ii) -*x* + 3/2, -*y* + 1, *z* - 1.

### S2. Experimental

The title compound was synthesized by utilizing a reported procedure (Revathi *et al.*, 2015). In a 250 ml round-bottomed flask, 130 ml of ethylmethylketone was added to 4-hydroxypiperidiene (0.04 mol) and stirred well. Triethylamine (0.04 mol) was then added and the mixture was stirred for 10 min. 4-Chlorobenzoyl chloride (0.04 mol) was added and the reaction mixture was stirred at room temperature for about 2 hr. A white precipitate of triethylammonium chloride was produced, which was filtered and the filtrate was evaporated to obtain the crude product, crystallization from ethylmethylketone gave colourless block-like crystals of the unexpected title adduct (yield: 88%).

### S3. Refinement

H atoms were positioned geometrically and treated as riding on their parent atoms and refined with, C—H distances of 0.93–0.98 Å, an O—H distance of 0.82 Å, with *U*_iso_(H)= 1.5 *U*_eq_(*C*-methyl), *U*_iso_(H)= 1.2*U*_eq_(C,*O*) for other H atoms. The value of the absolute structure parameter (Parsons *et al.*, 2013), although of no relevance for the present structure was determined as 0.03 (3) using 583 quotients [(I+)-(I-)]/[(I+)+(I-)].

### Crystal data

**Table:**

0.75C_12_H_14_ClNO_2_·0.25C_12_H_14_ClNO	*D*_x_ = 1.312 Mg m^−^^3^
*M**_r_* = 235.69	Mo *K*α radiation, λ = 0.71073 Å
Orthorhombic, *P**c**a*2_1_	Cell parameters from 3452 reflections
*a* = 24.312 (4) Å	θ = 2.8–23.2°
*b* = 6.1628 (10) Å	µ = 0.30 mm^−^^1^
*c* = 7.9654 (11) Å	*T* = 293 K
*V* = 1193.5 (3) Å^3^	Block, colourless
*Z* = 4	0.25 × 0.20 × 0.20 mm
*F*(000) = 496	

### Data collection

**Table:**

Bruker Kappa APEXII CCD diffractometer	1539 reflections with *I* > 2σ(*I*)
Radiation source: Sealed tube	*R*_int_ = 0.031
ω and φ scan	θ_max_ = 26.1°, θ_min_ = 3.1°
Absorption correction: multi-scan (*SADABS*; Bruker, 2004)	*h* = −30→30
*T*_min_ = 0.930, *T*_max_ = 0.941	*k* = −7→7
17321 measured reflections	*l* = −9→9
2356 independent reflections	

### Refinement

**Table:**

Refinement on *F*^2^	Secondary atom site location: difference Fourier map
Least-squares matrix: full	Hydrogen site location: inferred from neighbouring sites
*R*[*F*^2^ > 2σ(*F*^2^)] = 0.048	H-atom parameters constrained
*wR*(*F*^2^) = 0.144	*w* = 1/[σ^2^(*F*_o_^2^) + (0.0664*P*)^2^ + 0.3379*P*] where *P* = (*F*_o_^2^ + 2*F*_c_^2^)/3
*S* = 1.02	(Δ/σ)_max_ < 0.001
2356 reflections	Δρ_max_ = 0.31 e Å^−^^3^
200 parameters	Δρ_min_ = −0.22 e Å^−^^3^
121 restraints	Absolute structure: Flack *x* determined using 583 quotients [(*I*^+^)-(*I*^-^)]/[(*I*^+^)+(*I*^-^)] (Parsons *et al.*, 2013)
Primary atom site location: structure-invariant direct methods	Absolute structure parameter: 0.03 (3)

### Special details

**Table:**

Geometry. All e.s.d.'s (except the e.s.d. in the dihedral angle between two l.s. planes) are estimated using the full covariance matrix. The cell e.s.d.'s are taken into account individually in the estimation of e.s.d.'s in distances, angles and torsion angles; correlations between e.s.d.'s in cell parameters are only used when they are defined by crystal symmetry. An approximate (isotropic) treatment of cell e.s.d.'s is used for estimating e.s.d.'s involving l.s. planes.

### Fractional atomic coordinates and isotropic or equivalent isotropic displacement parameters (Å^2^)

**Table:**

	*x*	*y*	*z*	*U*_iso_*/*U*_eq_	Occ. (<1)
C1	0.73230 (19)	0.1884 (8)	0.7580 (6)	0.0743 (13)	
C2	0.7397 (2)	0.3762 (10)	0.6747 (8)	0.0909 (16)	
H2	0.7748	0.4191	0.6416	0.109*	
C3	0.6943 (2)	0.5050 (9)	0.6390 (7)	0.0832 (15)	
H3	0.6991	0.6353	0.5819	0.100*	
C4	0.64254 (19)	0.4423 (8)	0.6867 (5)	0.0656 (12)	
C5	0.63637 (19)	0.2510 (8)	0.7729 (8)	0.0811 (14)	
H5	0.6016	0.2076	0.8082	0.097*	
C6	0.6815 (2)	0.1230 (8)	0.8074 (8)	0.0890 (16)	
H6	0.6771	−0.0076	0.8643	0.107*	
C7	0.5944 (2)	0.5838 (9)	0.6575 (6)	0.0773 (14)	
C8	0.5243 (3)	0.7357 (12)	0.4730 (9)	0.0749 (18)	0.75
H8A	0.5155	0.8165	0.5739	0.090*	0.75
H8B	0.4918	0.6565	0.4381	0.090*	0.75
C9	0.5419 (3)	0.8877 (11)	0.3372 (9)	0.0681 (17)	0.75
H9A	0.5727	0.9741	0.3766	0.082*	0.75
H9B	0.5119	0.9855	0.3109	0.082*	0.75
C10	0.5587 (3)	0.7668 (13)	0.1804 (9)	0.0711 (19)	0.75
H10	0.5263	0.6897	0.1375	0.085*	0.75
C11	0.6028 (3)	0.5988 (12)	0.2233 (8)	0.0690 (16)	0.75
H11A	0.6102	0.5109	0.1249	0.083*	0.75
H11B	0.6365	0.6732	0.2537	0.083*	0.75
C12	0.5859 (3)	0.4567 (10)	0.3627 (8)	0.0640 (14)	0.75
H12A	0.5554	0.3660	0.3269	0.077*	0.75
H12B	0.6162	0.3627	0.3935	0.077*	0.75
N1	0.5694 (2)	0.5841 (9)	0.5067 (6)	0.0659 (14)	0.75
O2	0.5746 (2)	0.8993 (9)	0.0685 (7)	0.0870 (14)	0.75
H2A	0.5924	0.8339	−0.0030	0.105 (17)*	0.75
C8'	0.5628 (8)	0.863 (3)	0.454 (3)	0.069 (4)	0.25
H8'1	0.5850	0.9758	0.4031	0.083*	0.25
H8'2	0.5466	0.9203	0.5556	0.083*	0.25
C9'	0.5169 (8)	0.795 (4)	0.331 (2)	0.072 (5)	0.25
H9'1	0.4939	0.9210	0.3107	0.087*	0.25
H9'2	0.4942	0.6872	0.3865	0.087*	0.25
C10'	0.5350 (9)	0.703 (4)	0.160 (2)	0.077 (6)	0.25
H10A	0.5559	0.8073	0.0953	0.093*	0.25
H10B	0.5040	0.6510	0.0946	0.093*	0.25
C11'	0.5714 (8)	0.515 (3)	0.226 (3)	0.069 (5)	0.25
H11C	0.5475	0.4128	0.2838	0.082*	0.25
H11D	0.5864	0.4396	0.1295	0.082*	0.25
C12'	0.6196 (7)	0.568 (3)	0.345 (2)	0.057 (4)	0.25
H12C	0.6383	0.4350	0.3775	0.069*	0.25
H12D	0.6458	0.6610	0.2887	0.069*	0.25
N1'	0.5977 (6)	0.677 (2)	0.4953 (15)	0.053 (3)	0.25
O1	0.57385 (17)	0.6828 (7)	0.7749 (5)	0.1119 (14)	
Cl1	0.78823 (7)	0.0274 (3)	0.7998 (3)	0.1274 (8)	

### Atomic displacement parameters (Å^2^)

**Table:**

	*U*^11^	*U*^22^	*U*^33^	*U*^12^	*U*^13^	*U*^23^
C1	0.067 (3)	0.080 (3)	0.076 (3)	0.011 (2)	−0.016 (2)	−0.004 (3)
C2	0.070 (3)	0.098 (4)	0.104 (4)	−0.010 (3)	0.001 (3)	0.003 (4)
C3	0.091 (4)	0.076 (3)	0.083 (4)	−0.003 (3)	−0.002 (3)	0.018 (3)
C4	0.073 (3)	0.071 (3)	0.053 (2)	0.012 (2)	0.002 (2)	0.010 (2)
C5	0.066 (3)	0.080 (3)	0.097 (3)	0.007 (3)	0.011 (3)	0.019 (3)
C6	0.092 (4)	0.072 (3)	0.103 (4)	0.017 (3)	0.001 (3)	0.023 (3)
C7	0.090 (3)	0.082 (3)	0.061 (2)	0.029 (3)	0.005 (2)	0.006 (2)
C8	0.074 (4)	0.076 (4)	0.075 (4)	0.031 (3)	0.005 (3)	0.011 (3)
C9	0.080 (5)	0.057 (3)	0.068 (4)	0.018 (3)	−0.012 (3)	0.004 (3)
C10	0.067 (4)	0.081 (4)	0.066 (3)	0.020 (3)	−0.018 (3)	−0.003 (3)
C11	0.067 (4)	0.082 (4)	0.058 (4)	0.020 (3)	−0.004 (3)	−0.004 (3)
C12	0.063 (3)	0.056 (3)	0.074 (3)	0.017 (3)	−0.010 (3)	−0.008 (3)
N1	0.072 (3)	0.064 (3)	0.062 (3)	0.023 (3)	−0.001 (2)	0.006 (2)
O2	0.097 (4)	0.088 (3)	0.076 (3)	0.017 (3)	0.004 (3)	0.004 (3)
C8'	0.079 (9)	0.064 (8)	0.064 (9)	0.032 (7)	−0.003 (8)	−0.001 (6)
C9'	0.067 (9)	0.080 (12)	0.071 (9)	0.034 (8)	−0.001 (7)	−0.007 (9)
C10'	0.057 (11)	0.085 (12)	0.090 (9)	0.012 (9)	0.012 (7)	−0.020 (8)
C11'	0.054 (8)	0.079 (10)	0.073 (9)	0.006 (7)	0.002 (7)	−0.023 (7)
C12'	0.050 (7)	0.059 (9)	0.062 (7)	0.009 (7)	0.001 (6)	−0.008 (6)
N1'	0.051 (7)	0.052 (6)	0.055 (4)	0.012 (5)	−0.004 (4)	−0.004 (4)
O1	0.121 (3)	0.147 (3)	0.067 (2)	0.057 (3)	0.008 (2)	−0.004 (3)
Cl1	0.0952 (10)	0.1290 (13)	0.1579 (17)	0.0462 (9)	−0.0304 (12)	−0.0134 (14)

### Geometric parameters (Å, º)

**Table:**

C1—C2	1.346 (7)	C10—H10	0.9800
C1—C6	1.358 (7)	C11—C12	1.472 (9)
C1—Cl1	1.716 (5)	C11—H11A	0.9700
C2—C3	1.390 (8)	C11—H11B	0.9700
C2—H2	0.9300	C12—N1	1.447 (8)
C3—C4	1.370 (7)	C12—H12A	0.9700
C3—H3	0.9300	C12—H12B	0.9700
C4—C5	1.373 (7)	O2—H2A	0.8200
C4—C7	1.478 (7)	C8'—N1'	1.460 (16)
C5—C6	1.378 (6)	C8'—C9'	1.54 (2)
C5—H5	0.9300	C8'—H8'1	0.9700
C6—H6	0.9300	C8'—H8'2	0.9700
C7—O1	1.223 (6)	C9'—C10'	1.540 (12)
C7—N1	1.346 (7)	C9'—H9'1	0.9700
C7—N1'	1.417 (13)	C9'—H9'2	0.9700
C8—N1	1.465 (7)	C10'—C11'	1.552 (12)
C8—C9	1.494 (9)	C10'—H10A	0.9700
C8—H8A	0.9700	C10'—H10B	0.9700
C8—H8B	0.9700	C11'—C12'	1.542 (19)
C9—C10	1.510 (9)	C11'—H11C	0.9700
C9—H9A	0.9700	C11'—H11D	0.9700
C9—H9B	0.9700	C12'—N1'	1.474 (16)
C10—O2	1.269 (9)	C12'—H12C	0.9700
C10—C11	1.529 (8)	C12'—H12D	0.9700
			
C2—C1—C6	121.4 (5)	C10—C11—H11B	109.2
C2—C1—Cl1	119.1 (4)	H11A—C11—H11B	107.9
C6—C1—Cl1	119.5 (4)	N1—C12—C11	110.6 (5)
C1—C2—C3	119.0 (5)	N1—C12—H12A	109.5
C1—C2—H2	120.5	C11—C12—H12A	109.5
C3—C2—H2	120.5	N1—C12—H12B	109.5
C4—C3—C2	120.8 (5)	C11—C12—H12B	109.5
C4—C3—H3	119.6	H12A—C12—H12B	108.1
C2—C3—H3	119.6	C7—N1—C12	125.6 (5)
C3—C4—C5	118.8 (4)	C7—N1—C8	120.2 (5)
C3—C4—C7	121.1 (4)	C12—N1—C8	114.1 (5)
C5—C4—C7	119.9 (4)	C10—O2—H2A	109.5
C4—C5—C6	120.3 (5)	N1'—C8'—C9'	110.8 (15)
C4—C5—H5	119.9	N1'—C8'—H8'1	109.5
C6—C5—H5	119.9	C9'—C8'—H8'1	109.5
C1—C6—C5	119.7 (5)	N1'—C8'—H8'2	109.5
C1—C6—H6	120.1	C9'—C8'—H8'2	109.5
C5—C6—H6	120.1	H8'1—C8'—H8'2	108.1
O1—C7—N1	119.8 (5)	C8'—C9'—C10'	116.9 (17)
O1—C7—N1'	121.2 (6)	C8'—C9'—H9'1	108.1
O1—C7—C4	119.8 (4)	C10'—C9'—H9'1	108.1
N1—C7—C4	119.9 (5)	C8'—C9'—H9'2	108.1
N1'—C7—C4	109.8 (6)	C10'—C9'—H9'2	108.1
N1—C8—C9	108.5 (6)	H9'1—C9'—H9'2	107.3
N1—C8—H8A	110.0	C9'—C10'—C11'	98.0 (15)
C9—C8—H8A	110.0	C9'—C10'—H10A	112.2
N1—C8—H8B	110.0	C11'—C10'—H10A	112.2
C9—C8—H8B	110.0	C9'—C10'—H10B	112.2
H8A—C8—H8B	108.4	C11'—C10'—H10B	112.2
C8—C9—C10	111.5 (6)	H10A—C10'—H10B	109.8
C8—C9—H9A	109.3	C12'—C11'—C10'	118.9 (17)
C10—C9—H9A	109.3	C12'—C11'—H11C	107.6
C8—C9—H9B	109.3	C10'—C11'—H11C	107.6
C10—C9—H9B	109.3	C12'—C11'—H11D	107.6
H9A—C9—H9B	108.0	C10'—C11'—H11D	107.6
O2—C10—C9	110.2 (6)	H11C—C11'—H11D	107.0
O2—C10—C11	112.2 (6)	N1'—C12'—C11'	108.7 (13)
C9—C10—C11	109.8 (5)	N1'—C12'—H12C	109.9
O2—C10—H10	108.2	C11'—C12'—H12C	109.9
C9—C10—H10	108.2	N1'—C12'—H12D	109.9
C11—C10—H10	108.2	C11'—C12'—H12D	109.9
C12—C11—C10	112.0 (6)	H12C—C12'—H12D	108.3
C12—C11—H11A	109.2	C7—N1'—C8'	119.5 (12)
C10—C11—H11A	109.2	C7—N1'—C12'	125.0 (12)
C12—C11—H11B	109.2	C8'—N1'—C12'	112.7 (13)
			
C6—C1—C2—C3	0.0 (8)	C10—C11—C12—N1	−53.4 (9)
Cl1—C1—C2—C3	179.1 (5)	O1—C7—N1—C12	−173.2 (6)
C1—C2—C3—C4	−0.3 (8)	C4—C7—N1—C12	−1.0 (10)
C2—C3—C4—C5	0.9 (8)	O1—C7—N1—C8	12.3 (10)
C2—C3—C4—C7	176.6 (5)	C4—C7—N1—C8	−175.4 (6)
C3—C4—C5—C6	−1.3 (8)	C11—C12—N1—C7	−116.7 (7)
C7—C4—C5—C6	−177.0 (5)	C11—C12—N1—C8	58.0 (9)
C2—C1—C6—C5	−0.3 (9)	C9—C8—N1—C7	115.9 (7)
Cl1—C1—C6—C5	−179.5 (5)	C9—C8—N1—C12	−59.2 (9)
C4—C5—C6—C1	1.0 (9)	N1'—C8'—C9'—C10'	−60 (3)
C3—C4—C7—O1	−104.8 (7)	C8'—C9'—C10'—C11'	56 (2)
C5—C4—C7—O1	70.8 (7)	C9'—C10'—C11'—C12'	−57 (2)
C3—C4—C7—N1	82.9 (7)	C10'—C11'—C12'—N1'	59 (2)
C5—C4—C7—N1	−101.4 (6)	O1—C7—N1'—C8'	−18.1 (18)
C3—C4—C7—N1'	42.4 (9)	C4—C7—N1'—C8'	−164.8 (13)
C5—C4—C7—N1'	−142.0 (8)	O1—C7—N1'—C12'	−177.3 (12)
N1—C8—C9—C10	56.9 (9)	C4—C7—N1'—C12'	36.0 (17)
C8—C9—C10—O2	−178.5 (7)	C9'—C8'—N1'—C7	−108.1 (19)
C8—C9—C10—C11	−54.4 (9)	C9'—C8'—N1'—C12'	54 (2)
O2—C10—C11—C12	175.3 (7)	C11'—C12'—N1'—C7	108.5 (19)
C9—C10—C11—C12	52.4 (9)	C11'—C12'—N1'—C8'	−52 (2)

### Hydrogen-bond geometry (Å, º)

**Table:**

*D*—H···*A*	*D*—H	H···*A*	*D*···*A*	*D*—H···*A*
O2—H2*A*···O1^i^	0.82	2.05	2.693 (7)	135
C12′—H12*D*···Cl1^ii^	0.97	2.77	3.63 (2)	148

Symmetry codes: (i) *x*, *y*, *z*−1; (ii) −*x*+3/2, *y*+1, *z*−1/2.
