# Supplementary figures and images for: Crystal structure of the adduct (4-chloro­phen­yl)(4-hy­droxy­piperidin-1-yl)methanone–(4-chloro­phen­yl)(piperidin-1-yl)methanone (0.75/0.25)
Source: Acta Crystallogr E Crystallogr Commun. 2015 Oct 31;71(Pt 11):o896–7. doi: 10.1107/S2056989015020265 (PMC4645011; doi:10.1107/S2056989015020265)

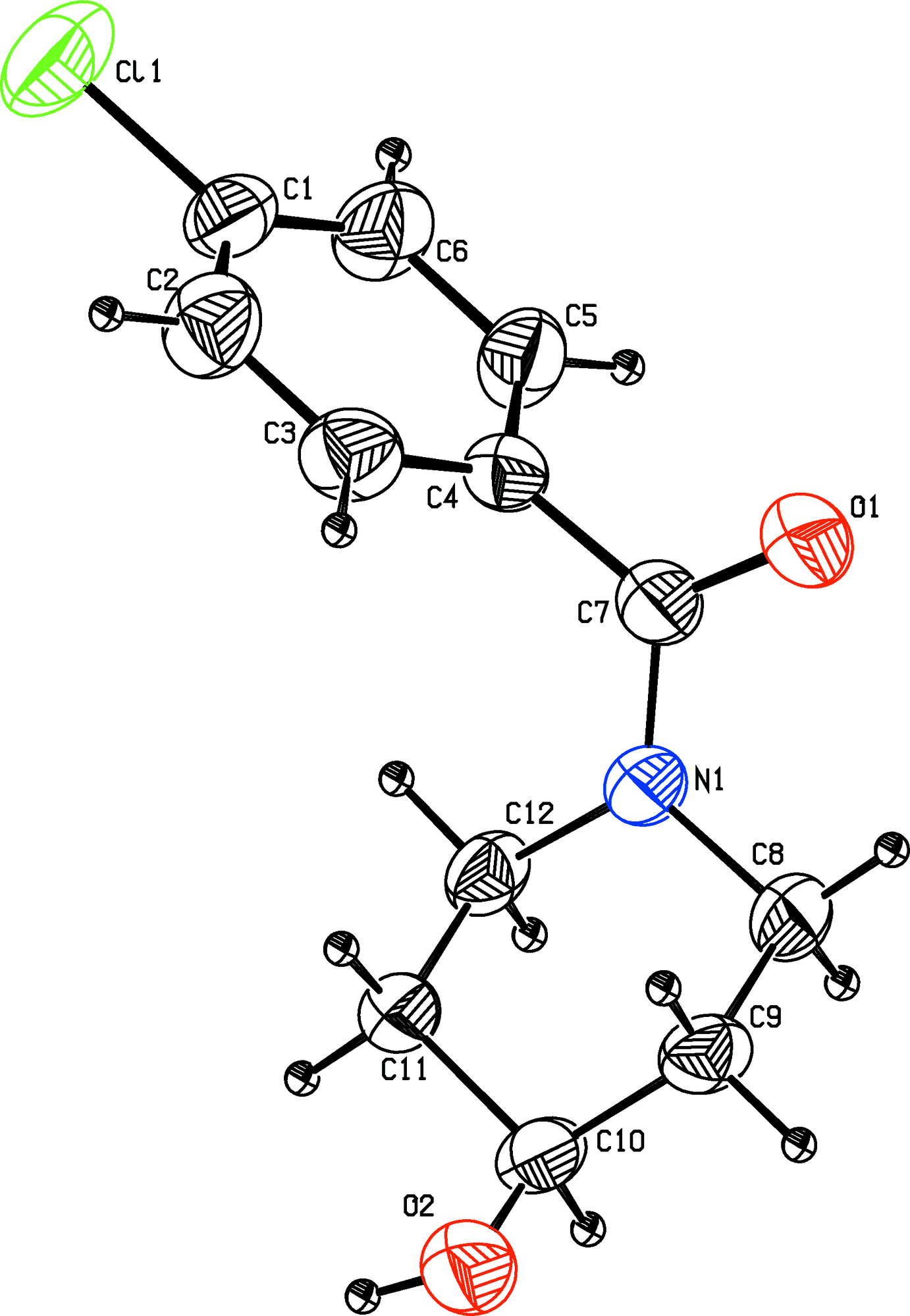

Supplement: Supplementary file 4 [file e-71-0o896-fig1.tif]

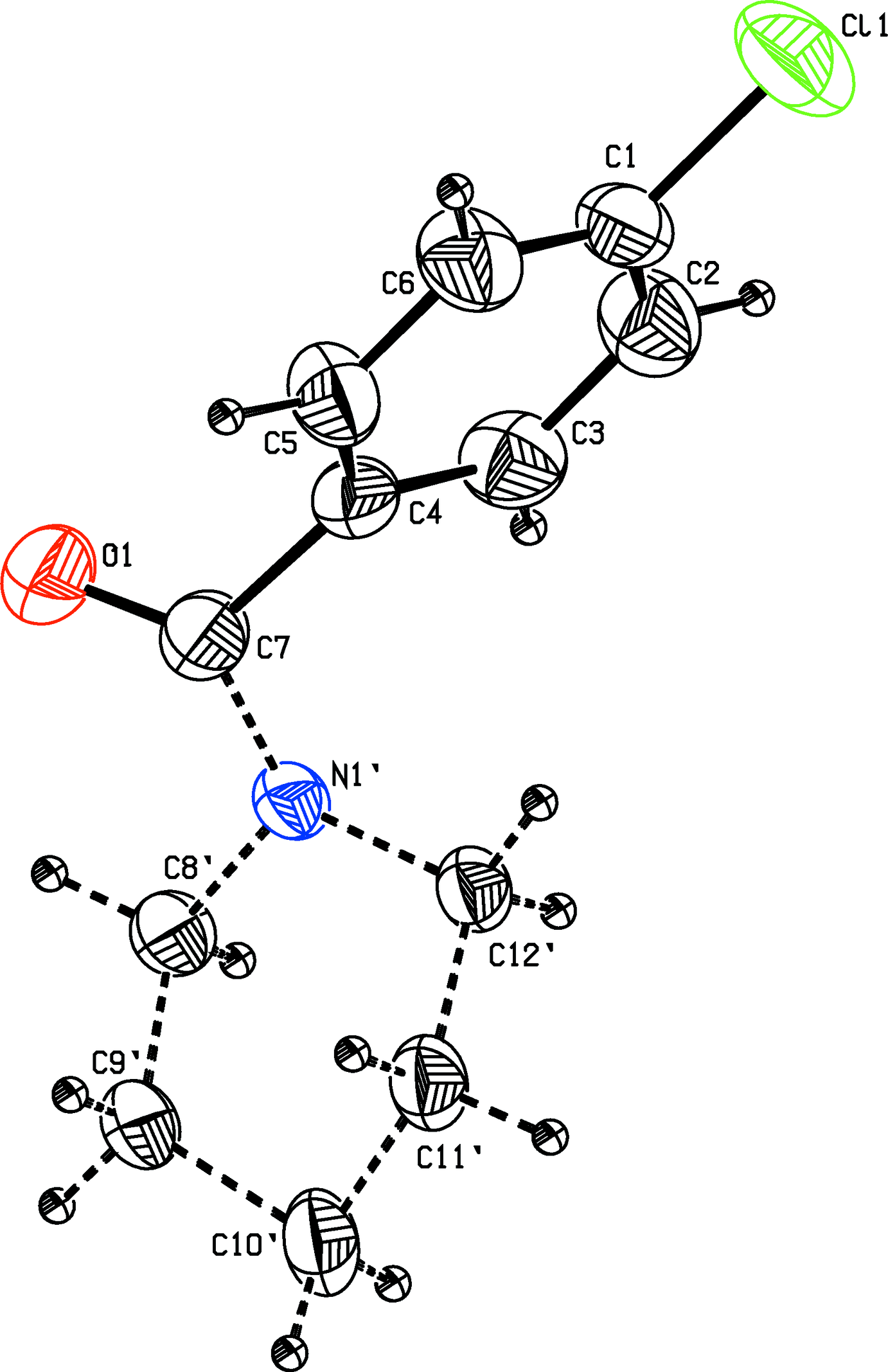

Supplement: Supplementary file 5 [file e-71-0o896-fig2.tif]

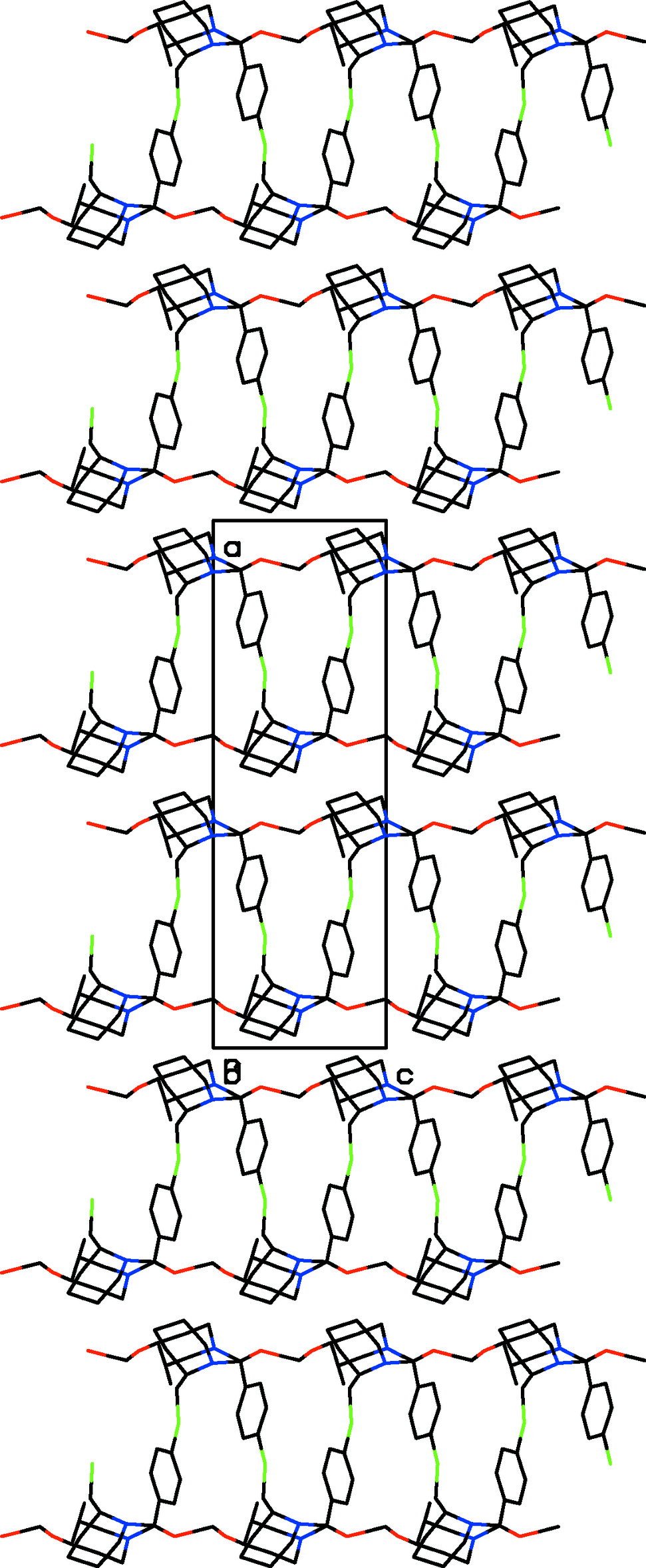

Supplement: Supplementary file 6 [file e-71-0o896-fig3.tif]
